# Adenocarcinoma of the appendix with metastasis to the spine

**DOI:** 10.4314/ahs.v24i4.30

**Published:** 2024-12

**Authors:** Emmanuel Olusola Oladeji, Peter Chinyere Osuala, Elizabeth Ayooluwa Idowu, Adefemi Oladiran Afolabi

**Affiliations:** 1 Department of Surgery, University College Hospital, Ibadan, Nigeria; 2 Department of Radiology, University College Hospital, Ibadan, Nigeria

**Keywords:** Adenocarcinoma, appendix, appendix carcinoma, bone metastasis, spine metastasis, Africa

## Abstract

There are a few reported cases of bone metastasis from adenocarcinoma of the appendix and there is none from Africa. We report this rare case of a 30-year-old man who presented with low back pain following a trivial fall and who subsequently developed generalized peritonitis for which he underwent laparotomy. The histology of the appendix and the mesentery confirmed adenocarcinoma of the appendix with metastasis to the mesenteric lymph nodes while an MRI scan confirmed spine metastasis. Carcinoma of the appendix, should, therefore, be considered as a differential diagnosis in spine metastasis of unknown origin especially with coexisting abdominal symptoms.

## Introduction

Since the first description of “appendiceal disease” by Jean Fernel in the mid-16^th^ century, and the first classic description of appendicitis about two centuries later by Lorenz Heister, the appendix has continued to occupy an important place in contemporary surgical practice.^1^-^3^ It is the most famous vestigial organ in the human body,^4^ and appendicectomy is one of the most performed procedures by general surgeons.^3^,^5^-^7^ Nevertheless, primary neoplasms of the appendix are not commonly encountered in surgical practice, they are found only in 0.5-1% of appendectomy specimens.^8^,^9^ Adenocarcinoma of the appendix is rare and has remained so since the first documented case by Beger in the late 19th century.^10^ It accounts for up to 60% of all appendiceal malignancies in some series, with most cases presenting after the 5^th^ decade of life.^8^,^11^ It presents most commonly with features of appendicitis and is hardly diagnosed preoperatively. Although there have been reports of axial bone metastasis from adenocarcinoma of the appendix,^12^,^13^ spine metastasis from adenocarcinoma of the appendix remains an extremely rare occurrence which, to the best of our knowledge, has not been reported previously in Africa. We present, therefore, a case of a 30-year-old man who was managed for adenocarcinoma of the appendix with spine metastasis.

## Case report

A 30-year-old male was admitted to the Emergency Department with a 5-week history of non-radiating moderately severe low back pain following a fall from a 2-metre height, and a 3-week history of painful progressive abdominal distension. The back pain had worsened with ambulation, with no associated lower limb weakness or sphincteric dysfunction. He had analgesics and application of a lumbar jacket and subsequently presented at the neurosurgical spine clinic where he was evaluated, and a request for Magnetic Resonance Imaging (MRI) was made. Three weeks before presentation at the Emergency Department, he developed generalized abdominal pain of insidious onset with associated progressive abdominal distention. There was associated constipation which was relieved by rectal digitation and enema. There was nausea but no vomiting, fever, or any other constitutional symptoms. He had no diagnosed co-morbidities, no remarkable social history, and no family history of malignancies.

**Fig. 1:**
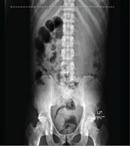
Abdominal x-ray showing relative gaslessness in the right iliac fossa and an adjacent dilated loop of bowel (white arrow)

Physical examination revealed a distended abdomen with generalized tenderness that was worse in the lower quadrants but without rebound tenderness or a palpable mass. The bowel sounds were absent. The anal sphincteric tone was normal and the rectum contained soft faeces. There was tenderness over the mid and lower lumbar spine but no gibbus, and motor and sensory examination in the lower limbs were normal. A clinical diagnosis of blunt abdominal injury with generalized peritonitis and traumatic lumbar spine injury was made. Abdominal X-rays (Fig 1) showed bilateral flank fullness, gaslessness in the right iliac fossa, and an adjacent dilated loop of bowel is seen extending from the right lumbar to the hypochondriac regions, suggestive of a sentinel loop. However, no significant air-fluid levels were seen. There were rectal gas shadows with multiple feacal balls seen within the rectum. An abdominal ultrasound scan showed marked ascites with internal multiple low-level echoes and increased cortical echogenicity with reduced corticomedullary differentiation of the kidneys, consistent with bilateral renal parenchymal disease.

**Fig. 2a:**
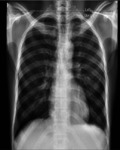
Chest x-ray showed normal findings. **Fig. 2b** Lateral lumbosacral x-ray (F-E study) showing lumbarisation of S1 vertebra with widespread radiolucent (black arrow) lesions within the vertebral bodies

**Fig. 3a:**
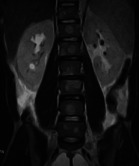
Sagittal T2W Thoracolumbar MRI and **Fig. 3b** Coronal T2W Lumbosacral MRI showing multiple widespread well-defined oval hyperintense (white arrows) lesions of varying sizes on MRI involving the whole spine

**Fig 4a:**
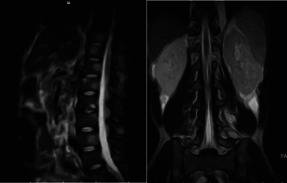
Sagittal T2W Lumbosacral MRI **Fig 4b** Coronal T2W Lumbosacral MRI showing multiple widespread well-defined oval hyperintense lesions (white arrows) involving the whole spine, sacrum, and iliac bones

Chest X-ray on admission (Fig. 2a) was normal. Plain lumbosacral X-rays (Fig. 2b) showed lumbarisation of the S1 vertebra with widespread radiolucent lesions of varying sizes within the vertebral bodies, sacrum, and iliac bones which were corroborated as multiple widespread well-defined oval hypointense (on T1W) and hyperintense (on T2W) lesions of varying sizes on MRI involving the whole spine, sacrum and iliac bone (Fig. 3a,3b, 4a, 4b)

Serum carcinoembryonic antigen was elevated (29.7ng/ml) while the urine was negative for Bence-Jones protein. Baseline complete blood count showed anaemia (haematocrit-29.5 %) and normal total leukocyte count (7,900/mm3) with relative monocytosis (15.5%). The serum electrolytes, urea, and creatinine were normal.

**Fig. 5:**
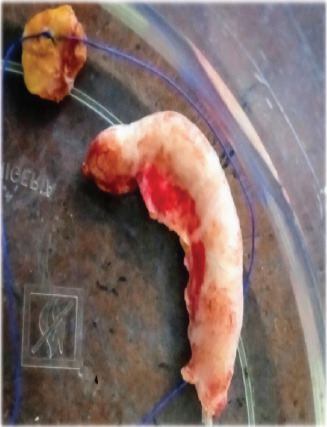
Excised cord-like appendix and a mesenteric lymph node (tagged with suture)

Laparotomy revealed 1.5-litre straw-coloured fluid, a greyish white, thickened cord-like retrocecal appendix with multiple mesenteric lymph nodes (Fig. 5) mostly in the ileocecal region. He had an appendectomy and mesenteric lymph node biopsy, and insertion of a pelvic drain.

**Fig. 6:**
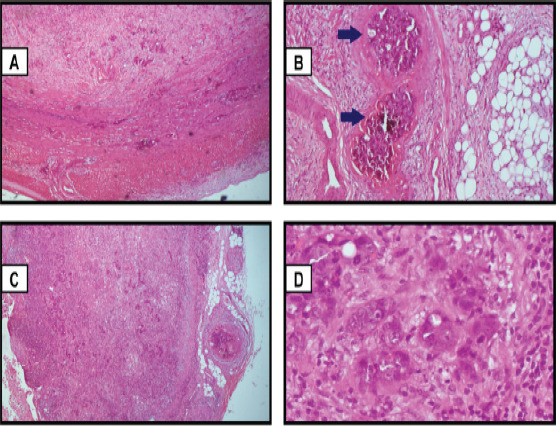
A: The appendix shows infiltration of the submucosa and muscularis (Hematoxylin and Eosin 40 X). B: The appendix with the arrows shows lymphovascular permeation of the serosa by tumour cells (Hematoxylin and Eosin 100 X). C: The lymph node showing total effacement of the nodal architecture with replacement by malignant epithelial neoplasm. (Hematoxylin and Eosin 40 X). D: Lymph node showing marked pleomorphism (Hematoxylin and Eosin 400 X)

The peritoneal fluid sample was negative for acid-fast bacilli (GeneXpert). Histology of the appendix specimen showed a malignant epithelial neoplasm composed of nests, cords, and tubules of atypical cells that have replaced a portion of the appendiceal mucosa with transmural infiltration and microscopic positive margin (Fig 6). Some of the cells showed intracytoplasmic mucin with a signet ring appearance. There was stromal desmoplasia with moderate to marked infiltration by acute and chronic inflammatory cells. These findings were consistent with an overall diagnosis of moderately differentiated carcinoma of the appendix with mesenteric lymph node metastasis. Our local oncology multidisciplinary team (MDT) made a diagnosis of metastatic adenocarcinoma of the appendix and recommended palliative chemotherapy and spine radiation.

Eight days post-surgery, he developed clinical and biochemical features of acute kidney injury necessitating two sessions of hemodialysis, with clinical improvement. He had repeated sessions of abdominal paracentesis for malignant ascites and was serially transfused with fresh frozen plasma. While he was being optimized for palliative chemotherapy and spine radiation, his clinical condition deteriorated precluding chemoradiation therapy. He was on terminal care till he died on the 25^th^ day of admission, from disease progression.

## Discussion

Adenocarcinoma of the appendix constitutes less than 0.5% of all gastrointestinal neoplasms and has an age-adjusted incidence of 0.95 per 1,000,000 population.^8^,^14^ It has a male preponderance and is mostly diagnosed after the 5^th^ decade of life,^8^,^14^-^16^ although it has been reported in lower age groups, including a 10-year-old.^17^ While the earlier seminal series documented carcinoid tumor as the appendiceal malignancy with the highest incidence,^10^,^18^,^19^ more recent population-based studies found adenocarcinoma to be the most common histologic type, with quoted figures as high as 60% in some series.^8^,^11^,^14^

Although most accessible histopathologic reviews of appendectomy specimens from Africa revealed no records of adenocarcinoma of the appendix,^5^-^7^,^20^-^22^ Edward et al recently reported a case of mucinous adenocarcinoma from Nigeria.^23^ Mbuk and Amber also found 1 out of the 124 retrospectively reviewed cases (which corresponds to 0.8%) of gastrointestinal tumors to be adenocarcinoma of the appendix.^24^ However, this report is the first case of adenocarcinoma of the appendix with spine metastasis to be reported from Nigeria. The only previously reported neoplasm of the appendix from our centre was a case of mucinous cystadenoma.^25^ Most patients with adenocarcinoma of the appendix present with features of acute appendicitis. Occasionally, the lesion is incidentally recognized following appendectomy for a different indication. Some patients may manifest non-specific abdominal symptoms or features of a tubo-ovarian disease while a few will present with tumor-related symptoms.^8^,^9^,^15^ The latter scenario is applicable to our patient, who had no abdominal symptoms before the fall. The clinical presentation of progressive abdominal pain which was preceded by a fall had led to a misdiagnosis of blunt abdominal injury with generalized peritonitis, and a traumatic lumbar spine injury.

Likewise on imaging, acute appendicitis from luminal obstruction is the most common manifestation for most tumor types, making it difficult to differentiate benign from malignant causes until histology is carried out. Other findings that may be encountered on imaging include intussusception, gastrointestinal bleeding, ureteral obstruction or hematuria, and increasing abdominal girth from rupture of a malignant mucocele, resulting in pseudomyxoma peritonei. Detection of these neoplasms at preoperative imaging is important because it may change the surgical approach and obviate additional surgery.^28^

The lumbosacral x-ray findings of multiple lytic lesions corroborated the spine MRI findings of florid hypointense lesions involving the entire spine which were suggestive of secondaries from an unknown primary mitotic lesion, but no suggestion of a likely appendiceal source. Cross-sectional imaging, particularly computed tomography (CT), is effective in the evaluation of these neoplasms. The typical finding includes an ill-defined soft tissue mass in the appendix, which may invade adjacent structures.^29^ The appearance of appendiceal adenocarcinoma with early obstruction is typically that of appendicitis, consisting of a thickened, inflamed appendix with non-filling with contrast material and peri-appendiceal fat stranding.^29^

Further assessment with Positron emission tomography (PET) and its combination with CT or MRI would have offered additional information on the molecular, functional, and morphological nature of the spine metastasis and the likely source of the primary mitotic lesion. However, our index patient was unable to undergo these investigations due to difficulties with timely access in our setting, which is a limitation of this case report. Intraoperative findings of widespread enlarged mesenteric lymph nodes which showed dominant presence in the ileocecal region suggested a diagnosis of abdominal tuberculosis which was however excluded by a Mycobacterium tuberculosis nucleic acid amplification test. Histology of the suspicious appendix and mesenteric lymph node biopsy specimens confirmed metastatic moderately differentiated adenocarcinoma of the appendix. Metastasis of appendiceal adenocarcinoma, seen in advanced disease, is mostly through the lymphatic route and is found in 23-37% of cases.^8^,^9^,^14^

The most common site is the peritoneum as seen in our patient, less commonly the liver and lungs. Even though the spine is a frequent site of metastasis for gastrointestinal cancers, with the mechanism of spread well elucidated in the literature,^26^,^27^ a primary appendiceal malignancy is seldom encountered. This may explain why such a diagnosis was not even remotely considered in this patient before surgery. Although it has been previously reported a few times in the literature,^12^,^13^, there is still a dearth of literature on the presentation and management of patients with spine metastasis from adenocarcinoma of the appendix. Synchronous gastrointestinal tumours have been found to occur in as much as 10-35% of cases^8^,^16^, but none was identified in this patient.

Our multidisciplinary team acknowledged that the clinical, intraoperative, imaging, and histopathologic findings in this patient correlate with a Duke stage III disease with metastasis to the spine. The range of appropriate treatment for this stage includes palliative appendectomy with adjuvant chemotherapy, use of cytoreductive surgery and perioperative intraperitoneal chemotherapy including Hyperthermic Intraperitoneal Chemotherapy (HIPEC), and postoperative irradiation. While some carefully selected patients with advanced disease may benefit from aggressive surgical intervention with right hemicolectomy and adequate lymph node dissection, the most superior option remains debatable.^8^,^9^,^14^ Unfortunately, the majority of the patients do not get the appropriate oncologic resection because the diagnosis is not usually made intraoperatively.^14^ Where there is an intraoperative suspicion of an appendiceal neoplasm, obtaining a frozen section of the appendix will be invaluable in deciding on the appropriate oncologic resection. This will not only reduce the incidence of reoperation for definitive surgery but also improve the overall patient outcome. Given the rarity of adenocarcinoma of the appendix, the options of chemotherapy regimen are largely informed by empirical evidence in the management of colorectal cancers, which include 5-fluorouracil-based treatment schedules, carboplatin with paclitaxel, and capecitabine with oxaliplatin.^14^-^16^ The role of other treatment modalities such as Vascular Endothelial Growth Factor (VEGF) inhibitors, and other molecularly targeted therapies remains controversial.^13^ This patient was being optimized for chemotherapy and spine irradiation until he had deterioration in his performance status.

Primary metastatic disease, as seen in this patient, is a poor prognostic factor. With a 5-year survival rate of 6-11%, the outcome is often estimated in weeks and months,^12^,^14^ as demonstrated in this patient who died from disease progression within 3 weeks of diagnosis. The late presentation, delay in diagnosis, as well as inadequate oncologic resection may have also contributed to the unfavourable outcome. Availability of frozen section for intraoperative diagnosis could have informed appropriate management decisions that might have improved the overall outcome.

## Conclusion

Adenocarcinoma of the appendix, even though rare, is one of the gastrointestinal malignancies that are hardly diagnosed preoperatively and has the potential for widespread metastases, including spread to the spine. It is therefore an important differential diagnosis to consider in the evaluation of a patient with spine metastasis of unknown origin especially with co-existing abdominal symptoms. With an intraoperative high level of suspicion of adenocarcinoma of the appendix, a frozen section is invaluable for decision-making to ensure an appropriate oncological resection is offered and a reoperation avoided.
